# Improved Mechanical Properties of Copoly(Phthalazinone Ether Sulphone)s Composites Reinforced by Multiscale Carbon Fibre/Graphene Oxide Reinforcements: A Step Closer to Industrial Production

**DOI:** 10.3390/polym11020237

**Published:** 2019-02-01

**Authors:** Nan Li, Xiuxiu Yang, Feng Bao, Yunxing Pan, Chenghao Wang, Bo Chen, Lishuai Zong, Chengde Liu, Jinyan Wang, Xigao Jian

**Affiliations:** State Key Laboratory of Fine Chemicals, Department of Polymer Science and Materials, School of Chemical Engineering, Dalian University of Technology, Dalian 116024, China; polymerlinan@dlut.edu.cn (N.L.); yxx1176364453@163.com (X.Y.); bfisvip@163.com (F.B.); yunwenyuluo@163.com (Y.P.); wangchh@mail.dlut.edu.cn (C.W.); chenbo011@163.com (B.C.); zongls@dlut.edu.cn (L.Z.); liucd@dlut.edu.cn (C.L.)

**Keywords:** carbon fibers, polymer-matrix composites (PMCs), interface/interphase

## Abstract

The properties of carbon fibre (CF) reinforced composites rely heavily on the fibre-matrix interface. To enhance the interfacial properties of CF/copoly(phthalazinone ether sulfone)s (PPBES) composites, a series of multiscale hybrid carbon fibre/graphene oxide (CF/GO) reinforcements were fabricated by a multistep deposition strategy. The optimal GO loading in hybrid fibres was investigated. Benefiting from the dilute GO aqueous solution and repeated deposition procedures, CF/GO (0.5%) shows a homogeneous distribution of GO on the hybrid fibre surface, which is confirmed by scanning electron microscopy, atomic force microscope, and X-ray photoelectron spectroscopy, thereby ensuring that its PPBES composite possesses the highest interlaminar shear strength (91.5 MPa) and flexural strength (1886 MPa) with 16.0% and 24.1% enhancements, respectively, compared to its non-reinforced counterpart. Moreover, the incorporation of GO into the interface is beneficial for the hydrothermal ageing resistance and thermo-mechanical properties of the hierarchical composite. This means that a mass production strategy for enhancing mechanical properties of CF/PPBES by regulating the fiber-matrix interface was developed.

## 1. Introduction

Carbon fibre (CF) with multiple high performance characteristics, such as high strength, high modulus, light weight, and erosion-resistance, have been widely used as ideal reinforcements for advanced composites in aviation, space flight, automobiles, and sports equipment [1,2]. It is generally accepted that the performance of the CF reinforced composites depends strongly on the fibre-matrix interface. Good interfacial properties are helpful for an efficient transfer of the load from the matrix to the fillers, which is essential to reduce stress concentrations and improve the mechanical properties [3,4]. However, owing to the nonpolar and inert fibre surface, the wettability and adsorption capacity of the CFs are poor [5]. Therefore, various CF surface treatment techniques involving the physical coating treatment [6,7] and chemical grafting methods [8,9] have been focused on to enhance the wettability of CF and the interfacial strength of the composites.

**Figure 1:**
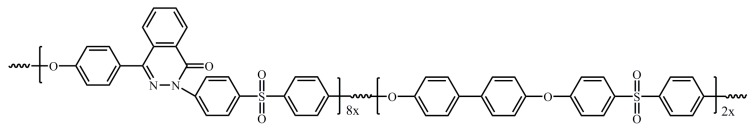
Molecular structure of PPBES.

As a particular type of engineering plastics, copoly(phthalazinone ether sulphone)s (PPBES) (Figure 1) are characterized by a favourable damage tolerance, excellent mechanical strength, and high thermal stability, and have been broadly used as structure materials for multitudinous application domains [10]. Compared to its counterparts, such as poly(ether ether ketone) (PEEK), polysulphone (PSU), and poly(phenylene sulphide) (PPS), the operating temperature for PPBES is 240 °C, which is superior to PSU (180 °C) and PPS (220 °C). Another advantage of PPBES is its good solubility in some common solvents, including N,N-dimethylacetamide, N-methyl-2-pyrrolidone, and chloroform, which permit the CF/PPBES composites to be manufactured through the solution impregnation technique [2]. However, PPS and PEEK composites can only be produced by melt impregnation, which requires the use of costly equipment and high energy consumption. The mechanical properties and thermal stability of PPBES composites can be significantly improved by the incorporation of CF into the resin. Owing to the large thermoplastic melt viscosity and the absence of chemical reactions during the hardening process, the interface adhesion between CF and PPBES is relatively weak. The poor interface greatly impedes the applications of CF/PPBES composites [11,12]. To improve the safety level and prolong the service life of PPBES composites, fiber surface treatment is indispensable to improve the surface wettability and mechanical interlocking between CF and PPBES [13].

**Figure 2:**
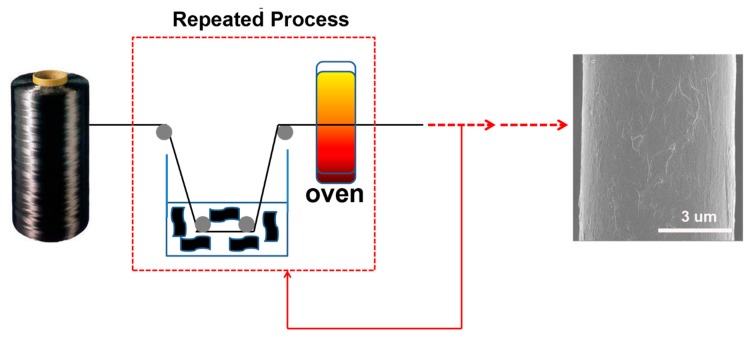
The schematic of the fabrication Carbon Fiber/Graphene Oxide hybrid fibers.

Recently, intense efforts were focused on assembling the nanoparticles onto CF to prepare multiscale reinforcements and enable their wide application for reinforcing polymer composites [14,15]. The great enhancement of interlaminar properties is closely related to the hierarchical reinforcing structures [13]. Graphene oxide (GO), a single layer of graphite oxide bearing oxygen functional groups on its basal planes and edges, has already attracted tremendous interest in the field of polymer composite science [16]. Compared to other carbon fillers, such as carbon nanofibres and carbon nanotubes, its high surface area, good wettability, low fabrication cost, and fascinating mechanical properties make GO the materials of choice for next-generation hierarchical reinforcements of polymer composites [17,18]. Recently, some studies have been devoted to chemically grafting the GO onto CF through the use of various bridging agents [13,19]. However, complicated multi-step chemical processes and high bridging agent costs limit the rate of their popularization. To overcome the two abovementioned shortcomings, several researchers have attempted to dissolve GO in water or to use deposition and slurry onto the CF surface for the fabrication of new hierarchical reinforcements [20,21]. However, challenges still remain, mainly arising from the poor solubility of monolayer graphene oxide. It is difficult to prepare suitable hierarchical reinforcement containing sufficient quantities of GO via a single deposition process. Increasing the concentration of graphene would cause GO aggregation in hierarchical reinforcements, which leads to fragile interfacial interactions between graphene nanosheets and fibres and further weakens the multi-scale enhancement effects [22]. The development of a direct and efficient strategy for the fabrication of CF/GO multiscale hybrid reinforcements would represent a significant advance. In this work, we develop an approach in which GO is dissolved in water at a dilute concentration, which can be repeatedly applied to the fibre surface as the deposition agent. More importantly, GO is evenly coated on the CF surface and the quantity is regulated by the deposition frequency. The typical procedure for the preparation of CF/GO hierarchical reinforcements is presented in Figure 2.

## 2. Experiment

### 2.1. Materials

PPBES (DHPZ:BP = 8:2) resin were purchased from Dalian Polymer New Material Co., Ltd., Liaoning, China. DHPZ is an abbreviation of “4-(4-Hydroxylphenyl)(2H)-phthalazin-1-one”, and BP is an abbreviation of “4,4’-Dihydroxybiphenyl”. Both DHPZ and BP are bisphenol monomers required for PPBES polymerization. The role of DHPZ is to increase the solubility of PPBES in DMAc, and the role of BP is to reduce the melt viscosity of PPBES. The solubility and melt viscosity of PPBES are two significant parameters in composite manufacturing based on the solution impregnation technique. CFs (T700, 12 K, average diameter 7 μm) were purchased from Toray Industries, Inc. Natural graphite particles with the average diameter of 50 μm were purchased from Tianheda Graphite Co., Ltd. (Qingdao, China). All solvents and other reagents were purchased from commercial sources and used as received.

### 2.2. Preparation of CF/GO Hierarchical Reinforcements

The CFs were refluxed with petroleum ether and acetone to eliminate the possible effects of commercial size from the ILSS enhancement in the system, named Untreated CF. First, graphite oxide was produced by the Hummers method followed by a washing process. Then, the resulting products were dispersed in deionized water to prepare a suspension of 0.2 g/L. Ultrasonication (1200 W, 2h) was used to exfoliate the graphite oxide to layered GO and form a homogeneous aqueous solution. Then, a custom-made small-scale deposition treatment processing line was used to prepare the CF/GO hierarchical reinforcements. Untreated CF tows were pulled through the aforementioned GO aqueous solution via a nip roller, and subsequently evaporated water at 120 °C for 2 min in a gas oven. Through experimental analysis and mathematical calculation, we found that each single deposition process increases the GO by 0.1 wt % in hierarchical reinforcements. Here, we prepared hierarchical reinforcements involving various GO contents (0.1 wt %, 0.3 wt %, 0.5 wt %, 0.8 wt %, 1.0 wt %) corresponding to the repetition of the abovementioned deposition process with various frequencies (1, 3, 5, 8, 10), named CF/GO (0.1%), CF/GO (0.3%), CF/GO (0.5%), CF/GO (0.8%), and CF/GO (1.0%), respectively.

### 2.3. Preparation of CF/PPBES Composites

CF/PPBES composites were fabricated through a solution impregnating technique described in our previous paper [23]. First, 18 g PPBES resin was dissolved in 100 mL N,N-dimethylacetamide (DMAc), and then CF tows were passed through the abovementioned solution and winded on the metal frame, and the residual DMAc was removed following a specific drying schedule (120 °C/10 min, 150 °C /10 min, 180 °C /10 min, 220 °C /10 min, 280 °C /10 min). The resulted unidirectional tapes were tailored to a certain size (100 mm × 60 mm). Finally, 15 tailored prepregs were compacted together via a specific moulding process. The volume percentages of CFs in the laminates were 58%–63% based on the ASTM D3171-15 standard.

### 2.4. Characterization

A Thermo Nicolet Nexus 470 Fourier transform infrared (FTIR) spectrometer was used to scan the specimens 64 times in the wavenumber range of 400–4000 cm^−1^ with the resolution of 2 cm^−1^. XPS (ESCALAB 220i-XL, VG, UK) equipped with a monochromatised Al Ka source (1486.6 eV) at the base pressure of 2 × 10^−9^ mbar was performed to detect the surface elements of the GO and CF/GO reinforcements.

Raman spectroscopy was performed on an Invia Renishaw 2000 spectrometer using an Ar^+^ laser (wavelength is 514.5 nm) at room temperature. Raman focusing and imaging was conducted using a confocal micro-scope with an objective of 50 × and the spot diameter of 1–2 μm.

Transmission electron microscopy (TEM) images were captured using a JEOL JEM-2010 operating at 200 kV. The GO thickness and the surface morphology of CF/GO hierarchical reinforcements were determined by a dimension icon atomic force microscope (AFM) (Bruker Dimension Icon-PT). Field-emission scanning electron microscopy (FE-SEM) images were taken on a Nova NanoSEM 450 after coating gold on the samples.

A dynamic contact angle metre and tensiometer (DCAT21, Data Physics Instruments, Germany) were used to characterize the dynamic contact angle and surface energy. Deionized water (γ^d^ = 21.8 mN m^−1^, γ = 72.8 mN m^−1^) and diiodomethane (γ^d^ = 50.8 mN m^−1^, γ= 50.8 mN m^−1^, 99% purity, Alfa Aesar, Ward Hill, NY, USA) were applied as the test liquids. The dispersive and polar components of the hybrid CFs were calculated according to Equations (1) and (2) [24,25]:

(1) $$\gamma_{l}(1+\cos\theta )=2{\left(\gamma_{l}^{p}\gamma_{f}^{p}\right)}^{1/2}+2{\left(\gamma_{l}^{d}\gamma_{f}^{d}\right)}^{1/2}$$

(2) $$\gamma_{f}=\gamma_{f}^{p}+\gamma_{f}^{d}$$

where $\gamma_{l}$, $\gamma_{l}^{d}$, and $\gamma_{l}^{p}$ are the surface tension of the test liquid, and its dispersive and polar components, respectively. At least 25 samples were tested for each specimen.

Three-point short-beam bending tests were performed to measure the interlaminar shear strength (ILSS) of the CF/PPBES composites according to the ISO 14130 standard. The specimen dimensions were 20 mm × 10 mm × 2 mm. The specimens were measured at the cross-head speed of 1 mm min^−1^. The ILSS values were calculated according to Equation (3):

(3) $$ILSS=\frac{3P_{b}}{4bh}$$

where *P_b_* is the maximum compression load at fracture (N), *b* is the width of the specimen (mm), and *h* is the thickness of the specimen (mm). At least six measurements were performed for each composite.

A universal testing machine (5569, Instron, Boston, Massachusetts, USA) was used to study the tensile strength of different CFs according to the ASTM D3379-75. The test was applied at the speed of 10 mm/min, and the gauge length of the sample was 15 mm. A total of 30 data points were collected and the results were analysed using a two-parameter Weibull model [26,27]. The calculation method is as follows:

(4) $$\sigma_{t}=4\frac{F_{b}}{\pi d^{2}}\times 10^{-9}$$

where $\sigma_{t}$ is the tensile strength, $F_{b}$ is the maximum load value at fracture of the fiber, and d is the diameter of a single fiber.

(5) $$F\left(\sigma \right)=1-exp[-L\left(\sigma /\sigma_{0}{)}^{m}\right]$$

(6) $$P=1-F\left(\sigma \right)=exp[-L\left(\sigma /\sigma_{0}{)}^{m}\right]$$

where *L* is the length of carbon fiber, σ_0_ is the Weibull scale parameter, *m* is the Weibull shape parameter, *F (σ)* is the failure probability, and *P* is the survival probability.

Evaluating the logarithm of Equation (6) as Equation (7):

(7) $$lnln[1/\left(1-F\left(\sigma \right)\right)]=mln\sigma +lnL-ln\sigma_{0}^{m}$$

(8) F(σ)=n/(N+1)

where *N* is the total number of testing fiber, and n is the total number of testing fiber below σ.

Plotting lnln[1/(1−F(σ))] against lnσ, we can calculate *m* and $\sigma_{0}$ by the slope and intercept.

The average tensile strength can be calculated by Equation (9):

(9) $$\bar{\sigma }=\sigma_{0}L^{-1/m}\Gamma \left(1+1/m\right)$$

where Γ is the Gamma function.

The flexural strength of the CF/PPBES composites was tested according to ASTM D790-10 using a three-point flexural test method. The specimens with the dimensions of 80 mm × 12.5 mm × 2 mm were tested at the crosshead movement rate of 2 mm/min, with the span of 64 mm. At least six measurements were performed for each composite.

Dynamic mechanical analysis (DMA) tests were carried out with a TA Q800 instrument at 1 Hz and the heating rate of 3 °C/min under a single cantilever mode. The test temperature ranged from 25 °C to 300 °C. The composite sample dimensions were 35 mm × 6 mm × 2 mm for the composite samples.

The hydrothermal ageing test of the specimens was performed using the following method: CF/PPBES composites panels were immersed in boiling water at 373 K for 48 h and then the specimens were cut from these panels and used for the testing. The hydrothermal ageing resistance was evaluated by tracing the changes in their ILSS and flexural strength results.

## 3. Results and Discussion

### 3.1. Preparation of GO

**Figure 3:**
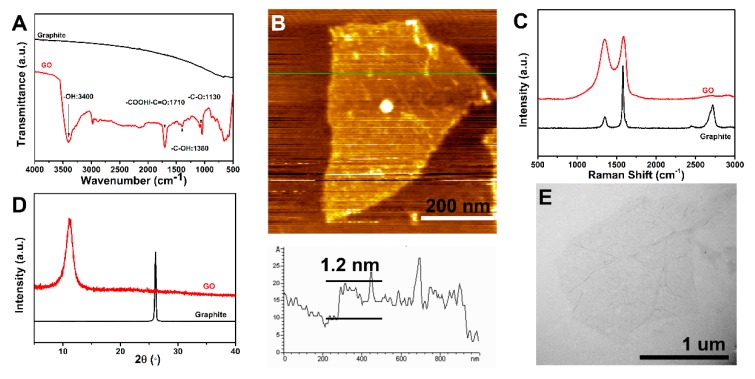
(**A**) FTIR spectra of GO and graphite, (**B**) AFM of GO, (**C**) Raman spectra of GO and graphite, (**D**) XRD spectra of GO and graphite, (**E**) TEM of GO.

The FTIR spectra of graphite and GO are presented in Figure 3A. While graphite contains no characteristic absorption peaks, GO contains a considerable amount of oxygen-containing functional groups. The band centred at approximately 3400 cm^−1^ corresponds to the stretching vibration of –OH groups. The characteristic peak at 1710 cm^−1^ is attributed to the stretching vibration of the –C=O bond in the –COOH group [28]. Moreover, the characteristic bands at 1380 cm^−1^ and 1130 cm^−1^ are related to the deformation vibration of the –C–OH group and the stretching vibration of the –C–O group, respectively [29]. These results demonstrate that GO contains a considerable amount of oxygen-based functional groups, which would promote the resins to wet the CF/GO hybrid fibres and improve the interface adhesion between the fibres and the matrix.

The chemical structures of graphite and GO are further confirmed by Raman spectra as seen from the results shown in Figure 3C. Two prominent peaks, corresponding to the D and G bands at 1350 cm^−1^ and 1590 cm^−1^, are clearly visible. For pristine graphite, the intensity ratio between the D-mode and G-mode (I_D_/I_G_) peaks is calculated to be 0.37, while the I_D_/I_G_ significantly increases to 0.95 for GO. These characteristic changes of Raman spectra indicate an increasing amount of sp3 carbon atoms’ formation in GO.

The XRD spectra of graphite and GO are shown in Figure 3D. A sharp characteristic diffraction peak at 2θ = 26.5° of graphite is related to the diffraction of the (002) plane, corresponding to the interlayer spacing of approximately 0.34 nm [30]. For GO, a broad diffraction peak at 2θ = 10.5° replaces the diffraction of the (002) plane in graphite, indicating a larger interlayer spacing (0.84 nm), owing to the incorporation of different oxygenic functional groups and structure defects by the Hummers method [29]. The difference in the interlayer spacing between graphite and GO demonstrates that graphite oxide has been exfoliated to produce layered GO nanosheets.

The morphology of GO was investigated by AFM (Figure 3B) and TEM (Figure 3E). The AFM image shows that the average height of GO is 1.2 nm, which is in good agreement with previous reported single-layer values [31]. TEM presents a quite thin wrinkled silk veil wavy morphology and the GO dimensions are aapproximately 1 μm ×1 μm. The unique crumpled structure of GO played a crucial role in improving the mechanical interlocking and the stress transfer between the hybrid fibres and matrix.

### 3.2. Surface Morphologies of CFs

**Figure 4:**
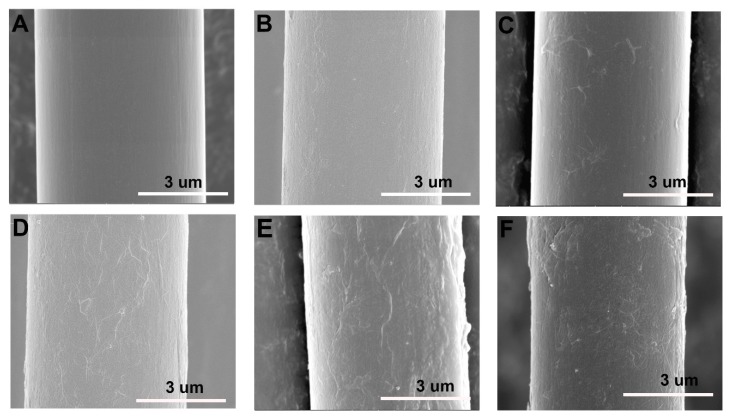
SEM images of (**A**) untreated CF, (**B**) CF/GO (0.1%), (**C**) CF/GO (0.3%), (**D**) CF/GO (0.5%), (**E**) CF/GO (0.8%), (**F**) CF/GO (1.0%).

The surface morphology evolution of CFs after different functionalization were observed by SEM, as shown in Figure 4. Obvious differences in the morphologies between untreated CF and hybrid fibres can be clearly observed. The untreated CF (Figure 4A) has a smooth and flat surface morphology. After coating by GO, it can be seen that a new hierarchical structure is formed. For CF/GO (0.1%) (Figure 4B), owing to the low GO content in hybrid fibre, no obvious graphene nanosheets can be observed on the fibre surface. Rather, only some wrinkled structures and tiny bulges are present. For CF/GO (0.3%) (Figure 4C), a few nanosheets are scattered over the CF surface and do not form a full coverage on the CFs, which affects the interface enhancement effect of GO. For CF/GO (0.5%) (Figure 4D), graphene nanosheets coat the CF evenly, similar to a georgette. The excellent flexibility of graphene creates considerable wrinkled structures on the fibre surface, which can improve the mechanical interlocking and load transfer between the fibres and the matrix [21]. For CF/GO (0.8%) (Figure 4E), massive bulges can be observed on the hybrid fibre surface, which is a signature of agglomeration caused by the higher concentration. For CF/GO (1.0%) (Figure 4F), the agglomeration of GO becomes significant, and excessive graphene nanosheets assemble into some clusters, which may form stress concentrations at the interface and weaken the interfacial strength [16].

**Figure 5:**
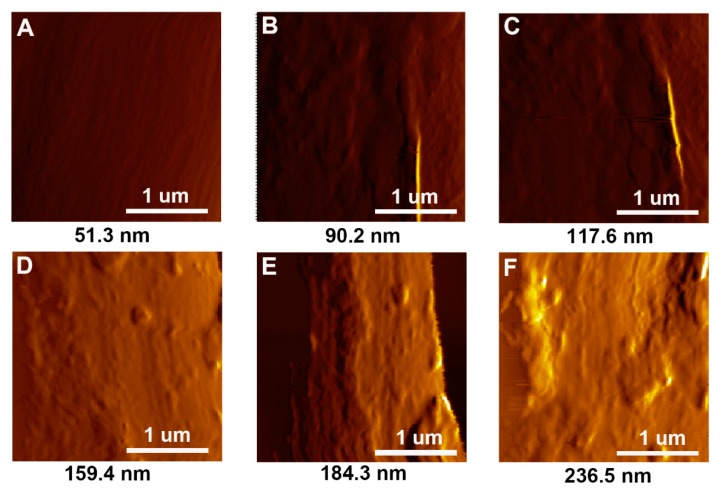
AFM images of (**A**) untreated CF, (**B**) CF/GO (0.1%), (**C)** CF/GO (0.3%), (**D**) CF/GO (0.5%), (**E**) CF/GO (0.8%), (**F**) CF/GO (1.0%).

The surface morphology of hybrid fibres was further investigated by AFM. The surface roughness test was also carried out by the AFM. The AFM images of different samples (Figure 5) show variations in the surface roughness, in agreement with the SEM observations. For untreated CF, the fibre surface is very smooth, and the surface roughness (*R*_a_ = 51.3 nm) is low. After coating with GO, an obvious improvement of the surface roughness can be observed. The surface roughness values for CF/GO (0.1%), CF/GO (0.3%), CF/GO (0.5%), CF/GO (0.8%), and CF/GO (1.0%) are 90.2 nm, 117.6 nm, 159.4 nm, 184.3 nm, and 236.5 nm, respectively. The surface roughness of the hybrid fibres increases with the GO content. It is generally accepted that the increased surface roughness is beneficial for improving the mechanical interlocking between the fibres and the matrix. However, excessive roughness probably means obvious agglomerations, and the surface roughness values of CF/GO (0.8%) (*R*_a_ = 184.3 nm) and CF/GO (1.0%) (*R*_a_ = 236.5 nm) are the result of agglomeration caused by the high GO content.

### 3.3. Surface Chemical Elemental Compositions of CFs

**Figure 6:**
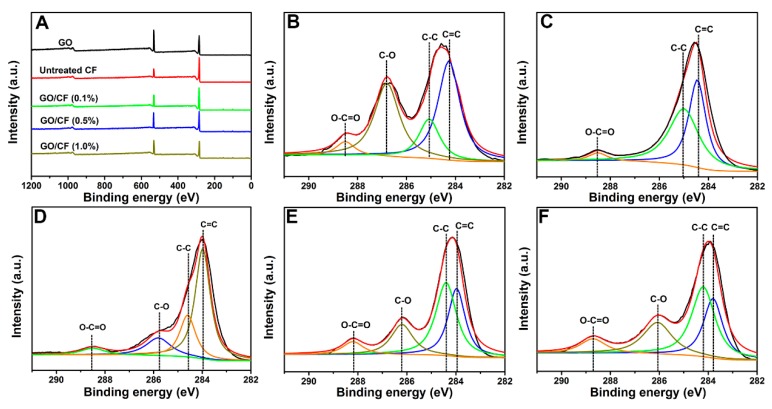
XPS curve fitting of (**A**) wide scan of GO and CFs, (**B**) C 1s peak of GO, (**C**) C 1s peak of CF/GO (0.1%), (**D**) C 1s peak of CF/GO (0.5%), (**E**) C 1s peak of CF/GO (1.0%).

The surface element compositions of GO, untreated CF, and hybrid CFs with 0.1 wt %, 0.5 wt %, and 1.0 wt % GO, respectively, were elucidated by XPS, and the results are shown in Figure 6. The full range XPS survey spectra (Figure 6A) demonstrates the existence of O1s (532 eV) and C1s (285 eV) [32]. The oxygen content of the CF/GO hybrid fibre surfaces increases with the GO content. According to Table 1, the O/C atomic ratio is 0.20 for untreated CF, 0.24 for CF/GO (0.1%), 0.32 for CF/GO (0.5%), 0.33 for CF/GO (1.0%), and 0.36 for pristine GO. The oxygen content of CF/GO (0.5%) is close to those of GO and CF/GO (1.0%), from which we can deduce two conclusions. On the one hand, hybrid CFs are nearly fully covered and wrapped by 0.5 wt % GO. On the other hand, GO is superfluous or even can be aggregated in CF/GO (0.8%) and CF/GO (1.0%) as seen from the results of Figure 4 and Figure 5.

The high resolution C1s XPS profiles (Figure 6B–F) were used to further study the chemical environment difference before and after the coating by GO. The C1s spectrum for untreated CF (Figure 6C) can be deconvoluted into three peaks at 284.0 eV, 285.0 eV, and 288.3 eV corresponding to sp2 hybridized carbon (C=C), sp3 hybridized carbon (C–C), and O–C=O, respectively [29]. However, another characteristic peak appearing at 286.5 eV in hybrid fibres is assigned to the C–O bonds corresponding to epoxide and hydroxyl originating from GO. The C–O ratio increases with GO content in hybrid fibres, and the peak intensity of the C–O groups of CF/GO (0.5%) is close to that of GO as shown in Figure 6E, indicating that hybrid fibres are nearly fully wrapped by GO.

### 3.4. Wettability of CFs

It is generally accepted that higher surface energy contributes to better wettability and stronger interfacial strength between the fibres and the matrix. The fibre surface energy depends on the surface chemical compositions and microstructures of the fibre. The advancing contact angle (θ), the surface energy (γ), dispersion component (γ^d^), and polar component (γ^p^) of different CFs are summarized in Table 2. For untreated CF, the surface free energy is only 42.01 mN/m owing to its non-polar and smooth graphitic surface. After coating by GO, the surface energy and its components increase significantly compared to those of untreated CF. Specifically, the γ^p^ increases with the GO content in hybrid CFs owing to an increased content of oxygen-containing functional groups. It is worth mentioning that the γ^d^ increases first and then decreases with increasing GO content. γ^d^ shows a peak value (43.09 mN/m) at 0.5 wt % GO loadings, indicating the appropriate surface microstructure. Unlike γ^d^, the γ^p^ for CF/GO (0.5%) (19.19 mN/m) is nearly equal to those for CF/GO (0.8%) (20.01 mN/m) and CF/GO (1.0%) (20.58 mN/m), which should be attributed to the similar O/C ratio. Compared to γ^p^, γd favours the enhancement of the surface energy. Hence, CF/GO (0.5%) with a high surface energy enhances the wettability effectively and thus improves the interfacial strength.

### 3.5. Composites Mechanical Property Testing

**Figure 7:**
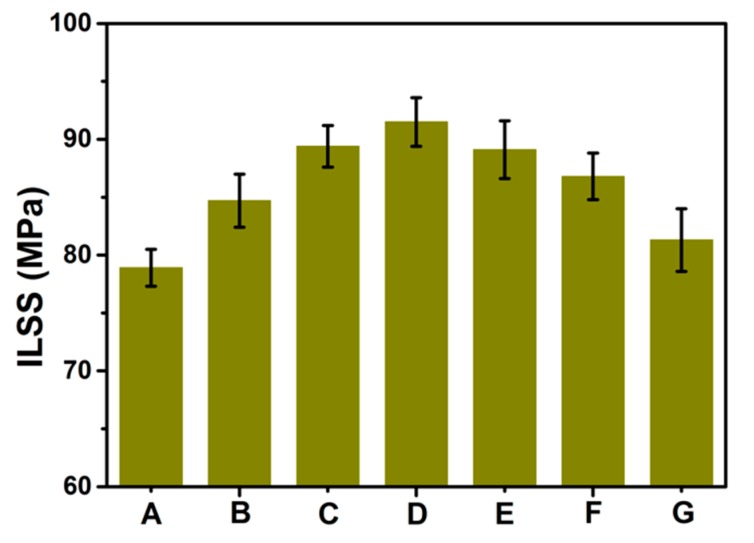
ILSS of composites: (**A**) Untreated CF, (**B**) CF/GO (0.1%), (**C**) CF/GO (0.3%), (**D**) CF/GO (0.5%), (**E**) CF/GO (0.8%), (**F**) CF/GO (1.0%), (**G**) CF/GO (0.5% one-step).

The ILSS of untreated CF and hybrid CFs reinforced PPBES is shown in Figure 7. The ILSS value of untreated CF composites is 78.9 MPa, and all composites with GO exhibit improved ILSS values compared to untreated CF. When the GO content is lower than 0.3 wt %, hierarchical reinforcements suffer from low GO content and lead to a limited improvement of the ILSS. As the GO loading is increased, a stable enhancement of the ILSS is observed. More importantly, the ILSS of the CF/GO (0.5%) composite is 16.0% higher than that of the baseline specimen. This improvement should be attributed to the increase of the surface energy and roughness of hybrid CF. However, when the GO content is beyond 0.8 wt %, the ILSS of hierarchical composites decreases slightly by increasing the GO content because a high loading of GO will lead to agglomeration in the interfacial region (as shown in Figure 4E) and induce stress concentration sites, weakening the energy dissipation and leading to a deterioration of the interfacial strength [33]. This conclusion is further supported by the decrease of ILSS for the CF/GO (1.0%) composite. Here, we developed a multistep deposition technique using an extremely dilute GO aqueous solution (0.2 g/L) to avoid agglomeration. To evaluate the advantages of this method, GO aqueous solution (0.8 g/L) deposition was applied to CF to directly prepare CF/GO (0.5%) hybrid fibres as a contrast experiment, named CF/GO (0.5% one-step). The ILSS of CF/GO (0.5% one-step) composite is 81.3 MPa, which is lower than that of the CF/GO (0.5%) composite (91.5 MPa). The advantages of this new technique for interfacial enhancement can be compared to the values reported in the literature. Fan et al. [6] reported a 12.7% improvement on the ILSS for GO-treated CF compared to the bare fibre composite. Xu et al. [16] reported a CF/GO hierarchical reinforcement containing 1.0 wt % GO, which is higher than the 0.5 wt % loading in this study. Zhao et al. [25] treated CFs with atmospheric helium plasma, and its epoxy composites showed a 7.1% improvement on ILSS. Wang et al. [34] treated CFs with polyether imide and graphene oxide complex deposition, and its PEEK composites exhibited a 12.0% enhancement for the ILSS. Compared to the previous reports, the surface modification method through repeatedly depositing dilute GO aqueous solution onto CFs also shows promising results for interface enhancement.

**Figure 8:**
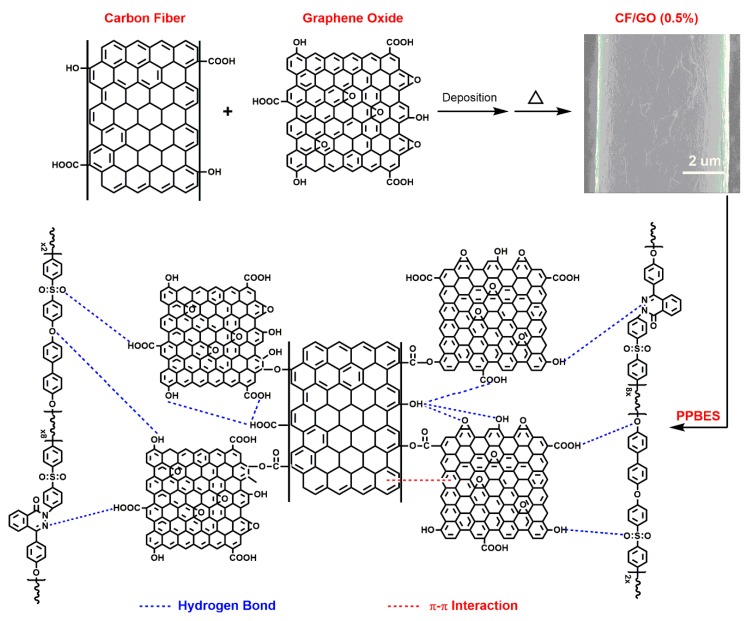
Schematic interface mechanism of CF/GO (0.5%) composites.

The interface mechanism between CF and GO, between CF/GO (0.5%) and PPBES are shown in Figure 8. A large number of oxygen-containing functional groups (e.g., –COOH, –OH) are implanted on the CF surface by the surface treatment technique during carbon fiber manufacturing. GO contains a considerable amount of epoxy groups, hydroxyl groups, and carboxyl groups. On the one hand, the -COOH and -OH on the CF surface can form chemical bonds and hydrogen bonds with the epoxy groups, hydroxyl groups, and carboxyl groups in GO. Besides, π-π interactions also exist between CF and GO. The chemical bonds, hydrogen bonds, and π-π interactions can enhance the adhesion between CF and GO. On the other hand, GO increases the number of –COOH and –OH on the CF/GO (0.5%) surface, which can form hydrogen bonds with the oxygen-based and nitrogen-based functional groups in PPBES chains. In conclusion, the chemical bonds, hydrogen bonds, and π-π interactions enhance the interfacial strength between CF/GO (0.5%) and PPBES.

**Figure 9:**
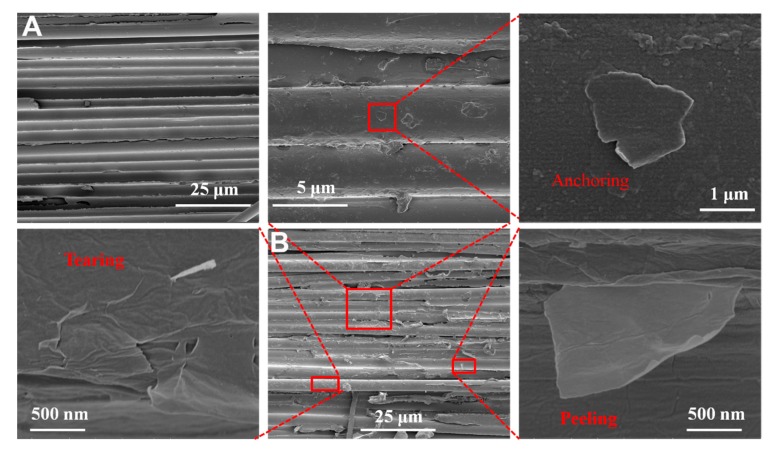
SEM morphologies of ILSS fracture surface of composite: (**A**) Untreated CF and (**B**) CF/GO (0.5%).

**Figure 10:**
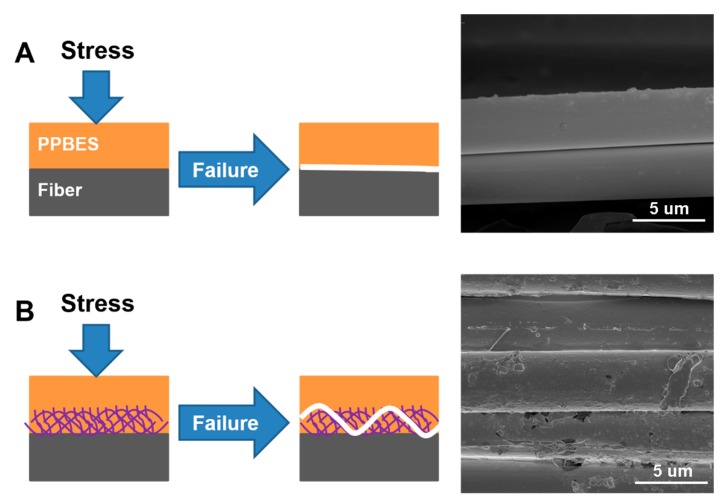
Schematic diagrams and micro-morphologies of the failure of composite interface of (**A**) untreated CF and (**B**) CF/GO (0.5%).

To study the reinforcement mechanism that leads to the enhanced interface properties, the fractured surface morphologies of untreated CF and CF/GO (0.5%) composites were observed by SEM, as shown in Figure 9. For untreated CF composite (Figure 9A), some bald CFs are exposed, indicating typical interface damage. For the CF/GO (0.5%) composite (Figure 9B), the GO coating on the fibres changes the surface microstructure and enlarges the surface area of the CF/GO (0.5%) reinforcement. Some PPBES wrapped by GO nanosheets are tightly anchored on CFs. GO acts as a button to bind the fibres and the matrix together via chemical bonds, hydrogen bonds, and π-π interactions, contributing to an improved ILSS. Moreover, some graphene nanosheets tear and peel from the CFs, bearing higher loads and dissipating additional energy. The failure of GO at the interface region may be induced by the local plasticisation and tear effect of the crack tip, preventing the generation and propagation of interface defects [35]. The schematic diagrams and micro-morphologies of the interface failure are shown in Figure 10. Untreated CF composite (Figure 10A) tends to fully separate from the fibre and retains a relatively clean fibre surface. However, the CF/GO (0.5%) composite (Figure 10B) exhibits a striking trans-GO layers pattern with a crack path that is not straight, and this disordered GO peeling/tearing may benefit the crack propagation resistance [35]. The random anchoring, peeling, and tearing of GO enhances the interface debonding resistance.

**Figure 11:**
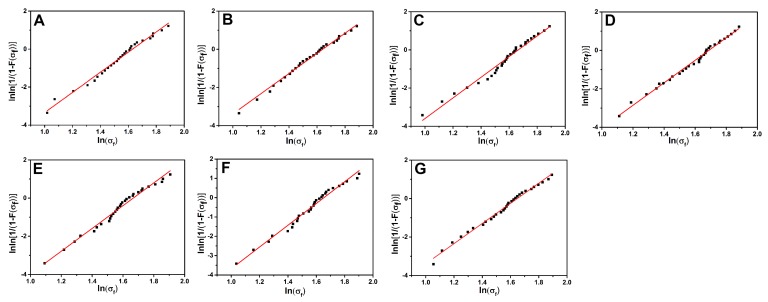
The Weibull distribution plots of carbon fibers: (**A**) Untreated CF, (**B)** CF/GO (0.1%), (**C**) CF/GO (0.3%), (**D**) CF/GO (0.5%), (**E**) CF/GO (0.8%), (**F**) CF/GO (1.0%), (**G**) CF/GO (0.5% one-step).

The mechanical strength of the CFRP composites depends strongly on the tensile strength (TS) of reinforcing materials. The TS of different CFs were characterized by the monofilament tensile test. Figure 11 shows the Weibull distribution plots of different CFs. The expectation values of TS calculated using the Weibull statistical method are presented in Table 3, where m and σ_0_ represent the Weibull shape parameter and scale parameter, respectively. The TS of CF/GO (0.1%), CF/GO (0.3%), CF/GO (0.5%), CF/GO (0.8%), CF/GO (1.0%), and CF/GO (0.5% one-step) are 4.76, 4.88, 5.03, 4.92, 4.84, and 4.78 GPa, respectively. All TS of hybrid fibres are higher than untreated CF (4.71 GPa). The graphene nanosheets wrap the CF surface randomly, possibly offering a strengthening mechanism by bridging the surface defects [22]. It is widely accepted that larger Weibull shape parameters correspond to smaller discreteness values. It is worth noting that the Weibull shape parameters of different reinforcements are related to the GO content. As the content of GO in multiscale reinforcements increases, the Weibull shape parameter first increases and then decreases. CF/GO (0.5) (5.85) and CF/GO (0.8%) (5.97) show relatively higher Weibull shape parameters than that of other reinforcements, which can be attributed to the saturated distribution of GO on CFs.

**Figure 12:**
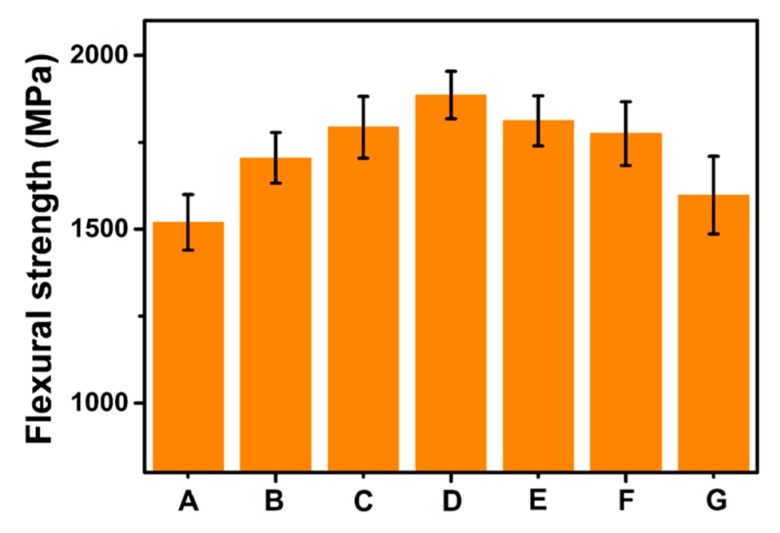
Flexural strength of composites: (**A**) Untreated CF, (**B**) CF/GO (0.1%), (**C**) CF/GO (0.3%), (**D**) CF/GO (0.5%), (**E**) CF/GO (0.8%), (**F**) CF/GO (1.0%), (**G**) CF/GO (0.5% one-step).

To further investigate the reinforcing effect of GO for interfacial properties, the flexural strength of the composites was studied, and the results are shown in Figure 12. It is clearly demonstrated that the flexural strengths of CF/GO hierarchical composites are greater than that of untreated CF composites. The strong interface after the incorporation of GO is beneficial for the effective transfer of the stress from the matrix to the reinforcements and leads to great flexural strength. However, superfluous GO nanosheets are prone to aggregate in the interfacial region and create new stress concentration sites to reduce the flexural strength [7]. The flexural strengths of the CF/GO hierarchical composites show a similar trend with the ILSS. The flexural strength of the CF/GO (0.5%) composite (1886 MPa) is 24.1% higher than that of the untreated CF composite. The large difference in the flexural properties between these two composites arises from the enhanced interface contributed by GO.

**Figure 13:**
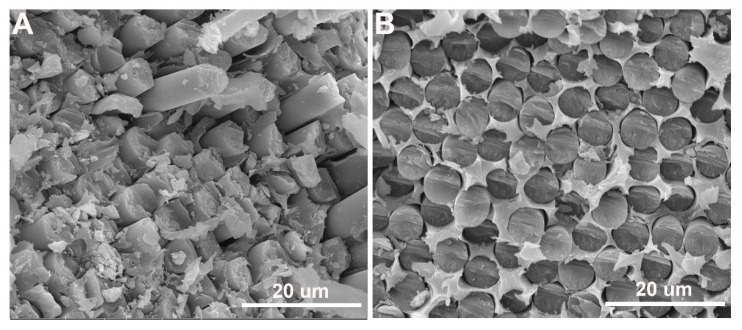
SEM morphologies of flexural fracture surface of composite: (**A**) Untreated CF and (**B**) CF/GO (0.5%).

The surface morphologies of untreated and CF/GO (0.5%) composite fractured surfaces after flexural tests were observed by SEM to analyse the interface reinforcement mechanism. Figure 13A shows the cross-section of the untreated CF composite. A large number of CFs was pulled out from PPBES, and the CF surfaces are extremely smooth, exhibiting apparent features of interface debonding. Additionally, many large holes remained in PPBES and are additional powerful evidence of interface debonding. The SEM image indicates a poor quality of interface between untreated CF and PPBES. However, for CF/GO (0.5%) composites (Figure 13B), interface debondings, holes, and pulled-out fibres were been observed owing to the incorporation of GO into the interface. The flexural fracture section is very compact, indicating better interface adhesion. The fracture section of CF/GO (0.5%) composites cracked by flexural stress demonstrates the strong interface bonding force between the fibres and the matrix.

### 3.6. Hydrothermal Ageing Performance of Composites

**Figure 14:**
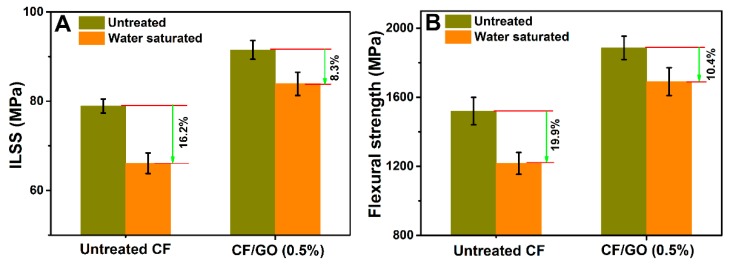
(**A**) ILSS and (**B**) flexural strength of untreated CF and CF/GO (0.5%) composites under saturated water absorption conditions.

Harsh environments with high humidity and temperature seriously threaten the fibre-matrix interface and thus degrade the safety of the composites. Hence, the ILSS and flexural strength of untreated CF and CF/GO (0.5%) composites after hydrothermal ageing tests were investigated, as illustrated in Figure 14. The ILSS and flexural strength of untreated CF composite suffering from hydrothermal ageing treatment decreases to 66.1 MPa and 1217 MPa, for apparent reductions of 16.2% and 19.9%, respectively. However, the ILSS and flexural strength of the CF/GO (0.5%) composite are 83.9 MPa and 1690 MPa, respectively, with decreases of only 8.3% and 10.4%, respectively. It is clear that GO nanosheets significantly enhance the hydrothermal ageing resistance of the composites. The weak interface of untreated CF composites facilitates the penetration of water molecules and produces many flaws at the interface region, followed by catastrophic interface failure. The hierarchical reinforcing interface of the CF/GO (0.5%) composite can reduce the stress concentration by improving the efficiency of stress transfer from PPBES to CFs and decrease the amount of the cracks, which can effectively protect the interface and prevent the composite from hydrothermal ageing failure [36].

### 3.7. Dynamic Mechanical Properties

**Figure 15:**
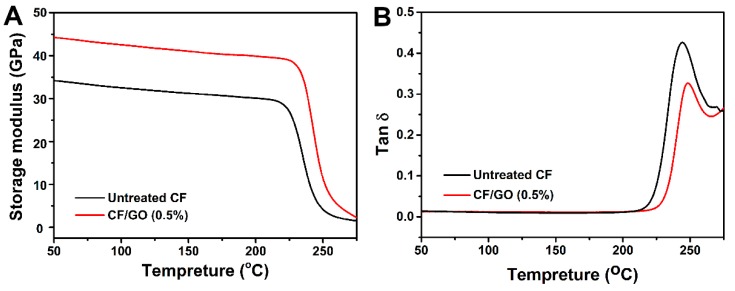
Temperature dependence of: (**A**) Storage modulus, and (**B**) tangent delta of composites.

Dynamic mechanical analysis was used to investigate the strength of the interaction between the polymer and the fibre. The effects of the GO nanosheets’ incorporation on the CF surface on the thermo-mechanical property of the composites are illustrated in Figure 15. From the storage modulus curves (Figure 15A), it can be seen that the glassy modulus increases from 34.5 GPa for the untreated CF composite to 44.5 GPa for the CF/GO (0.5%) composite. On the one hand, the presence of GO on the hybrid fibre enhances the wettability and mechanical interlocking between CFs and PPBES and results in a better interfacial adhesion, which can increase the volume fraction of the interphase in the composites and constrain the polymer chain mobility at the interphase region. This interface phase could serve as a reinforcement in the composite. On the other hand, previous research showed that the tensile modulus of GO is 207.6 GPa [37], which is higher than that of the PPBES resin, so that the GO could act as an additional reinforcement in the composite. The synergistic effect of these two factors stiffens the CF/GO (0.5%) composite [14].

The damping property of a composite depends on the balance between the viscous phase and elastic phase. A better interface can sustain stronger stress and dissipate less energy [12]. Conversely, a composite with weak interface adhesion is prone to dissipate more energy and exhibits a high damping peak value in the tan δ curves. In Figure 15B, the CF/GO (0.5%) composite shows a lower peak value of tan δ than that of the untreated CF composite, indicating a better interface property. Moreover, the peak temperature of the tan δ curve is used to define the glass transition temperature (*T_g_*). The *T_g_* for the CF/GO (0.5%) composite is approximately 250 °C, which is approximately 8 °C higher than that of the untreated CF composite. The better interface restricts the PPBES chains’ mobility at the interface region and results in a high *T_g_*. The increased storage modulus, *T_g_*, and decreased tan δ peak value can be attributed to the improved interface adhesion of the CF/GO (0.5%) composite [38].

## 4. Conclusions

In summary, we explored a novel strategy for the fabrication of CF/GO hybrid fibres through repeated deposition of dilute GO aqueous solution onto CFs. This approach is simple and feasible and may be applied to industrial fabrication of CF/GO hybrid reinforcement to enhance the interface property of its composites. We systematically investigated the effects of GO content (deposition frequency) in hybrid fibres on the mechanical properties of the composites. Owing to a combination of surface microstructure, roughness, and oxygen content, the CF/GO (0.5%) composite showed the highest ILSS (91.5 MPa) and flexural strength (1886 MPa) with a 16.0% and 24.1% increase, respectively, compared to the untreated CF composite (78.9 MPa and 1520 MPa, respectively). The enhanced wettability and mechanical interlocking caused by GO improves the interface adhesion between the hybrid fibres and the matrix. Additionally, GO can passivate the crack tip and change the crack path, thereby preventing crack propagation through its failure (tearing and peeling). Furthermore, the insertions of GO also improve the hydrothermal ageing resistance and dynamic thermo-mechanical property of hierarchical composites. This study may shed some light on the mass production of multiscale reinforcements and their application in the aerospace and automotive industries.

## Figures and Tables

**Table 1:** Surface element analysis of GO and CFs.

Samples	GO	Untreated CF	CF/GO (0.1%)	CF/GO (0.5%)	CF/GO (1.0 %)
C (%)	73.33	83.35	80.72	75.64	74.96
O (%)	26.67	16.65	19.28	24.36	25.04
O/C	0.36	0.20	0.24	0.32	0.33

**Table 2:** Dynamic contact angles and surface energies of different reinforcements.

Sample	Contact Angle θ (°)		γ^d^ (mN/m)	γ^p^ (mN/m)	γ (mN/m)
	Water	Diiodomethane			
Untreated CF	69.3	55.2	31.33	10.68	42.01
CF/GO (0.1%)	63.8	48.3	35.21	12.29	47.50
CF/GO (0.3%)	57.2	40.1	39.56	14.32	53.88
CF/GO (0.5%)	46.1	32.6	43.09	19.19	62.28
CF/GO (0.8%)	46.8	38.3	40.47	20.01	60.48
CF/GO (1.0%)	47.2	41.7	38.76	20.58	59.34

**Table 3:** The Weibull shape parameter (m), scale parameter (σ_0_), and single fiber tensile strength of carbon fiber specimens.

Samples	m	σ_0_	Expectation (GPa)
Untreated CF	5.31	2.44	4.71
CF/GO (0.1%)	5.28	2.46	4.76
CF/GO (0.3%)	5.40	2.56	4.88
CF/GO (0.5%)	5.85	2.77	5.03
CF/GO (0.8%)	5.97	2.75	4.92
CF/GO (1.0%)	5.64	2.61	4.84
CF/GO (0.5%-one step)	5.24	2.46	4.78

## References

[1] Li N., Wu Z.Q., Yang X.X., Wang C.H., Zong L.S., Pan Y.X., Wang J.Y., Jian X.G. (2018). One-pot strategy for covalent construction of POSS-modified silane layer on carbon fiber to enhance interfacial properties and anti-hydrothermal aging behaviors of PPBES composites. J. Mater. Sci..

[2] Li N., Zong L.S., Wu Z.Q., Liu C., Wang X., Bao F., Wang J.Y., Jian X.G. (2017). Amino-terminated nitrogen-rich layer to improve the interlaminar shear strength between carbon fiber and a thermoplastic matrix. Compos. Part A Appl..

[3] Wang Z.K., Xian G.J., Zhao X.L. (2018). Effects of hydrothermal aging on carbon fibre/epoxy composites with different interfacial bonding strength. Constr. Build. Mater..

[4] Semitekolos D., Kainourgios P., Jones C., Rana A., Koumoulos E.P., Charitidis C.A. (2018). Advanced carbon fibre composites via poly methacrylic acid surface treatment; surface analysis and mechanical properties investigation. Compos. Part B Eng..

[5] Gao B., Zhang R.L., He M.S., Wang C.G., Liu L., Zhao L.F., Wen Z.J., Ding Z.P. (2018). Interfacial microstructure and mechanical properties of carbon fiber composites by fiber surface modification with poly (amidoamine)/polyhedral oligomeric silsesquioxane. Compos. Part A Appl..

[6] Zhang X.Q., Fan X.Y., Yan C., Li H., Zhu Y.D., Li X.T., Yu L.P. (2012). Interfacial microstructure and properties of carbon fiber composites modified with graphene oxide. ACS Appl. Mater. Interfaces.

[7] Chen L., Jin H., Xu Z.W., Shan M.J., Tian X., Yang C.Y., Wang Z., Chen B.W. (2014). A design of gradient interphase reinforced by silanized graphene oxide and its effect on carbon fiber/epoxy interface. Mater. Chem. Phys..

[8] Yang X.B., Jiang X., Huang Y.D., Guo Z.H., Shao L. (2017). Building Nanoporous Metal-Organic Frameworks “Armor” on Fibers for High-Performance Composite Materials. ACS Appl. Mater. Interfaces.

[9] Wu G.S., Ma L.C., Wang Y.W., Liu L., Huang Y.D. (2016). Interfacial properties and impact toughness of methylphenylsilicone resin composites by chemically grafting POSS and tetraethylenepentamine onto carbon fibers. Compos. Part A Appl..

[10] Li N., Wu Z.Q., Huo L., Zong L.S., Guo Y.J., Wang J.Y., Jian X.G. (2016). One-step functionalization of carbon fiber using in situ generated aromatic diazonium salts to enhance adhesion with PPBES resins. RSC Adv..

[11] Yuan H.J., Zhang S.C., Lu C.X. (2014). Surface modification of carbon fibers by a polyether sulfone emulsion sizing for increased interfacial adhesion with polyether sulfone. Appl. Surf. Sci..

[12] Zhang K., Zhang G., Liu B.Y., Wang X.J., Long S.R., Yang J. (2014). Effect of aminated polyphenylene sulfide on the mechanical properties of short carbon fiber reinforced polyphenylene sulfide composites. Compos. Sci. Technol..

[13] Zhang C.X., Wu G.S., Jiang H. (2018). Tuning interfacial strength of silicone resin composites by varying the grafting density of octamaleamic acid-POSS modified onto carbon fiber. Compos. Part A Appl..

[14] Zhao F., Huang Y.D., Liu L., Bai Y.P., Xu L.W. (2011). Formation of a carbon fiber/polyhedral oligomeric silsesquioxane/carbon nanotube hybrid reinforcement and its effect on the interfacial properties of carbon fiber/epoxy composites. Carbon.

[15] Sui X.H., Shi J., Yao H.W., Xu Z.W., Chen L., Li X.J., Ma M.J., Kuang L.Y., Fu H.J., Deng H. (2017). Interfacial and fatigue-resistant synergetic enhancement of carbon fiber/epoxy hierarchical composites via an electrophoresis deposited carbon nanotube-toughened transition layer. Compos. Part A Appl..

[16] Chen L., Jin H., Xu Z.W., Li J.L., Guo Q.W., Shang M.J., Yang C.Y., Wang Z., Mai W., Chen B.W. (2015). Role of a gradient interface layer in interfacial enhancement of carbon fiber/epoxy hierarchical composites. J. Mater. Sci..

[17] Zhang R.L., Gao B., Ma Q.H., Zhang J., Cui H.Z., Liu L. (2016). Directly grafting graphene oxide onto carbon fiber and the effect on the mechanical properties of carbon fiber composites. Mater. Des..

[18] Hu Z., Shao Q., Moloney M.G., Xu X.R., Zhang D.Y., Li J., Zhang C.H., Huang Y.D. (2017). Nondestructive functionalization of graphene by surface-initiated atom transfer radical polymerization: An ideal nanofiller for poly(p-phenylene benzobisoxazole) fibers. Macromolecules.

[19] Li Y.B., Peng Q.Y., He X.D., Hu P.A., Wang C., Shang Y.Y., Wang R.G., Jiao W.C., Lv H.Z. (2012). Synthesis and characterization of a new hierarchical reinforcement by chemically grafting graphene oxide onto carbon fibers. J. Mater. Chem..

[20] Jiang D.W., Liu L., Wu G.S., Zhang Q.B., Long J., Wu Z.J., Xie F., Huang Y.D. (2017). Mechanical properties of carbon fiber composites modified with graphene oxide in the interphase. Polym. Compos..

[21] Li F., Liu Y., Qu C.B., Xiao H.M., Hua Y., Sui G.X., Fu S.Y. (2015). Enhanced mechanical properties of short carbon fiber reinforced polyethersulfone composites by graphene oxide coating. Polymer.

[22] Wang C.F., Li J., Yu J.L., Sun S.F., Li X.Y., Xie F., Jiang B., Wu G.S., Yu F., Huang Y.D. (2017). Grafting of size-controlled graphene oxide sheets onto carbon fiber for reinforcement of carbon fiber/epoxy composite interfacial strength. Compos. Part A Appl..

[23] Li N., Zong L.S., Wu Z.Q., Liu C., Wang J.Y., Jian X.G. (2017). Effect of Poly (phthalazinone ether ketone) with amino groups on the interfacial performance of carbon fibers reinforced PPBES resin. Compos. Sci. Technol..

[24] Wu G.S., Ma L.C., Jiang H., Liu L., Huang Y.D. (2017). Improving the interfacial strength of silicone resin composites by chemically grafting silica nanoparticles on carbon fiber. Compos. Sci. Technol..

[25] Sun J.F., Zhao F., Yao Y., Jin Z., Liu X., Huang Y.D. (2014). High efficient and continuous surface modification of carbon fibers with improved tensile strength and interfacial adhesion. Appl. Surf. Sci..

[26] Jiang B., Zhang T., Zhao L.W., Huang Y.D. (2017). Interfacially reinforced carbon fiber composites by grafting modified methylsilicone resin. Compos. Sci. Technol..

[27] Weibull W. (1951). A statistical distribution function of wide applicability. J. Appl. Mech..

[28] Zhou C.J., Wang S.F., Zhuang Q.X., Han Z.W. (2008). Enhanced conductivity in polybenzoxazoles doped with carboxylated multi-walled carbon nanotubes. Carbon.

[29] Hu Z., Li N., Li J., Zhang C.H., Song Y.J., Li X.L., Wu G.S., Xie F., Huang Y.D. (2015). Facile preparation of poly(p-phenylene benzobisoxazole)/graphene composite films via one-pot in situ polymerization. Polymer.

[30] Li Y.W., Li J., Song Y.J., Hu Z., Zhao F., Huang Y.D. (2013). In situ polymerization and characterization of graphene oxide-co-poly(phenylene benzobisoxazole) copolymer fibers derived from composite inner salts. J. Polym. Sci. Pol. Chem..

[31] Stankovich S., Dikin D.A., Dommett G.H.B., Kohlhaas K.M., Zimney E.J., Stach E.A., Richard D.P., Sonbinh T.N., Rodney S.R. (2006). Graphene-based composite materials. Nature.

[32] Hu F.Y., Wang J.Y., Hu S., Li L.F., Wang G., Qiu J.S., Jian X.G. (2016). Inherent N, O-containing carbon frameworks as electrode materials for high-performance supercapacitors. Nanoscale.

[33] Du S.S., Li F., Xiao H.M., Li Y.Q., Hu N., Fu S.Y. (2016). Tensile and flexural properties of graphene oxide coated-short glass fiber reinforced polyethersulfone composites. Compos. Part B Eng..

[34] Chen J.L., Wang K., Zhao Y. (2018). Enhanced interfacial interactions of carbon fiber reinforced PEEK composites by regulating PEI and graphene oxide complex sizing at the interface. Compos. Sci. Technol..

[35] Wang P.F., Yang J.L., Liu W.S., Tang X.Z., Zhao K., Lu X.H., Xu S.L. (2017). Tunable crack propagation behavior in carbon fiber reinforced plastic laminates with polydopamine and graphene oxide treated fibers. Mater. Des..

[36] Wu G.S., Chen L., Liu L. (2017). Direct grafting of octamaleamic acid-polyhedral oligomeric silsesquioxanes onto the surface of carbon fibers and the effects on the interfacial properties and anti-hydrothermal aging behaviors of silicone resin composites. J. Mater. Sci..

[37] Suk J.W., Piner R.D., An J., Ruoff R.S. (2010). Mechanical properties of monolayer graphene oxide. ACS Nano.

[38] Jiao W.W., Liu W.B., Yang F., Jiang L., Jiao W.C., Wang R.G. (2017). Improving the interfacial property of carbon fiber/vinyl ester resin composite by grafting modification of sizing agent on carbon fiber surface. J. Mater. Sci..

